# Sodium Channel *Na*_*v*_*1.5* Controls Epithelial-to-Mesenchymal Transition and Invasiveness in Breast Cancer Cells Through its Regulation by the Salt-Inducible Kinase-1

**DOI:** 10.1038/s41598-019-55197-5

**Published:** 2019-12-09

**Authors:** Frédéric Gradek, Osbaldo Lopez-Charcas, Stéphanie Chadet, Lucile Poisson, Lobna Ouldamer, Caroline Goupille, Marie-Lise Jourdan, Stéphan Chevalier, Driffa Moussata, Pierre Besson, Sébastien Roger

**Affiliations:** 1 EA4245 Transplantation, Immunologie, Inflammation; Université de Tours, Tours, France; 2 Inserm UMR1069, Nutrition, Croissance et Cancer; Université de Tours, Tours, France; 3 CHRU de Tours, Tours, France; 4 Institut Universitaire de France, Paris, France

**Keywords:** Breast cancer, Cell invasion, Breast cancer

## Abstract

Loss of epithelial polarity and gain in invasiveness by carcinoma cells are critical events in the aggressive progression of cancers and depend on phenotypic transition programs such as the epithelial-to-mesenchymal transition (EMT). Many studies have reported the aberrant expression of voltage-gated sodium channels (Na_V_) in carcinomas and specifically the Na_V_1.5 isoform, encoded by the *SCN5A* gene, in breast cancer. Na_V_1.5 activity, through an entry of sodium ions, in breast cancer cells is associated with increased invasiveness, but its participation to the EMT has to be clarified. In this study, we show that reducing the expression of Na_V_1.5 in highly aggressive human MDA-MB-231 breast cancer cells reverted the mesenchymal phenotype, reduced cancer cell invasiveness and the expression of the EMT-promoting transcription factor *SNAI1*. The heterologous expression of Na_V_1.5 in weakly invasive MCF-7 breast cancer cells induced their expression of both *SNAI1* and *ZEB1* and increased their invasive capacities. In MCF-7 cells the stimulation with the EMT-activator signal TGF-β1 increased the expression of *SCN5A*. Moreover, the reduction of the salt-inducible kinase 1 (SIK1) expression promoted Na_V_1.5-dependent invasiveness and expression of EMT-associated transcription factor SNAI1. Altogether, these results indicated a prominent role of SIK1 in regulating Na_V_1.5-dependent EMT and invasiveness.

## Introduction

The development of secondary tumours, also called metastases, in organs distant from the primary solid tumour, represents the ultimate level of cancer aggressiveness, associated with more than 90% of patient deaths^1^. At the cancer cell level, critical steps in the metastatic cascade involve morphological and functional changes, such as the loss of cell-cell junctions, loss of apicobasal epithelial polarity, and therefore the loss of the vectorial transport of ions and molecules across the epithelium, towards the acquisition of an elongated, spindle-shaped, “mesenchymal” phenotype. These changes in cell morphologies are associated with the gain of invasive capacities^2^, allowing carcinoma cells to penetrate surrounding tissues and intravasate into the lymphatic or blood circulation. These steps are parts of the multistage epithelial-to-mesenchymal transition (EMT) which promotes cancer cell dissemination from the primary tumour, while the reverse process, called mesenchymal-to-epithelial transition (MET) is proposed to support the outgrowth of tumoral foci in distantly colonized organs^3^. EMT is a very plastic programme reminiscent from trans-differentiation processes physiologically participating to tissue constitution during embryogenesis and tissue repair. In these physiological contexts, EMT/MET are tightly and timely regulated, and participate to the organogenesis and at maintaining organ functions. In the tumour context, multiple environmental conditions can induce EMT, such as hypoxia or paracrine signals coming from both stromal and immune cells (e.g. transforming growth factor beta, TGF-β1). The majority of these signals converge and act through the induction of EMT–associated transcription factors such as zinc finger proteins from the SNAI family, zinc finger and homeodomain proteins from the ZEB family or basic-loop helix proteins of the TWIST family^4^. It is well characterized that microenvironmental conditions in the tumour might induce plasticity programs in cancer cells and contribute to the tumour progression^5,6^. This might be the case of the ionic composition and concentration of the extracellular content, which could participate in signalling pathways promoting cancer cell proliferation, dedifferentiation and invasiveness. As such, early studies questioned the relationships between the Na^+^ content, cell permeability to Na^+^ and the consequences on malignant cell proliferation, invasive capacities and the development of metastases^7^. Indeed, energy-dispersive X-ray microanalyses recorded higher concentrations of Na^+^ in tumour cells comparatively to non-cancer cells^8^. These initial measurements were confirmed later on, using non-invasive ^23^Na-magnetic resonance imaging and an increased concentration of Na^+^ in malignant breast tumours was identified compared to surrounding normal tissues^9–11^, suggesting that this parameter could represent an indicator of malignancy, as well as a predictive marker of breast cancer response to the chemotherapy treatment^12,13^. This increase in Na^+^ concentration is very likely to concern the intracellular compartment of cancer cells^9^, in which it could modulate a whole set of signalling pathways. Salt-inducible kinase 1 (SIK1) is a serine/threonine kinase of the AMP-activated protein kinase (AMPK) family, originally cloned from adrenocortical glands of rats receiving a high salt diet^14^, which takes part into a sodium-sensing intracellular network regulating sodium content^15,16^. In breast cancer, a reduced expression of SIK1 has been associated with metastatic progression and with a poor outcome^17^. Furthermore, silencing SIK1 was demonstrated to prevent p53-mediated anoikis and to promote cancer cell colony growth and metastases development^18^, suggesting that SIK1 could act as a tumour-suppressor^19^. Moreover, knocking down SIK1 increased the migration of gastric adenocarcinoma cells^20^. SIK1 expression was significantly lower in hepatocellular carcinoma, compared to normal liver biopsies, and its overexpression in cancer cells suppressed the expression of EMT markers, tumour growth and metastases in a xenograft tumour model^21^.

Voltage-gated sodium channels (Na_V_) are multimeric transmembrane complexes composed of one large pore-forming principal subunit (9 genes *SCN1A-SCN5A*, *SCN8A-SCN11A* encoding 9 proteins, Na_V_1.1–1.9)^22,23^ and one or two smaller transmembrane subunits considered as auxiliary (4 genes *SCN1B* to *SCN4B*, generating five subunits, β1, β1B, β2, β3 and β4, which all possess a single membrane-spanning domain with the exception of β1B)^24^. The activity of Na_V_ generally gives rise to rapid and transient depolarizing Na^+^ currents (I_Na_) that are responsible for the generation of action potentials in excitable cells^25^. For this reason, Na_V_ have been considered as characteristic features of excitable cells. However, it has been shown that these channels are not only physiologically expressed in excitable cells, but also in normal immune and microglial cells, in which they participate to their activation and migration in response to environmental signals of threat^26–30^.

Multiple studies have also demonstrated the expression of Na_V_ in non-excitable cancer cells, while they are not expressed in normal cells, suggesting that they could be associated with dedifferentiation of transformed cells and more generally with carcinogenesis and cancer progression^31–33^. In a wide range of cancer cell lines and primary cultures of carcinomas, Na_V_ channels appeared to be fully functional at the plasma membrane, giving rise to recordable sodium currents, and to regulate cellular functions related to the invasive capacity^31,34–40^. Specifically, the Na_V_1.5 isoform (usually characterized as being the main cardiac isoform) which is the product of the *SCN5A* gene, was found to be highly overexpressed at both mRNA and protein levels in breast tumours, compared to normal tissues, and was correlated with cancer recurrence, metastases development and reduced patients survival^41–43^. In animal models of mammary cancer, the expression of Na_V_1.5 in breast cancer cells enhanced primary tumour growth and metastases development, and this was reduced in presence of pharmacological inhibitors of Na_V_^44,45^.

The activity of Na_V_1.5, resulting in the persistent entry of Na^+^ at the basal membrane potential (“window” current), was demonstrated in highly aggressive MDA-MB-231 human breast cancer cells, in which it was promoting extracellular matrix degradation and cancer cell invasiveness^46,47^. The activity of the channel is critical, since its inhibition using small molecules reduces extracellular matrix invasion^48^. In comparison, and while *SCN5A* was expressed at the mRNA level, no transient sodium current could be recorded in non-tumoural immortalized MCF-10A mammary cells, or even in weakly invasive and poorly dedifferentiated MCF-7 cancer cells^42,47,49^. Similar results were obtained in the context of non-small cell lung cancer cells, for which Na_V_ activity was recorded in several cancer cell lines such as H460, H23 and Calu-1, but not in non-cancer lung epithelial cells BEAS-2B and NL-20. In lung cancer cells, Na_V_ activity resulted in increases of intracellular sodium concentration and invasiveness^35^.

In breast cancer cells, the Na^+^ influx mediated through non-inactivated Na_V_1.5 channels was demonstrated to allosterically increase the activity of the Na^+^-H^+^ exchanger NHE1, thus promoting the efflux of H^+^ and further increasing the entry of Na^+^ into cancer cells, subsequently alkalinizing the intracellular pH and lowering the extracellular pH^47,49,50^. The acidification of the pericellular microenvironment was demonstrated to be favourable to the activity of extracellular proteases digesting the extracellular matrix, such as acidic cysteine cathepsins, thus allowing invasion of the extracellular matrix by cancer cells^47,49–51^. Furthermore, Na_V_1.5 activity was shown to sustain Src kinase activity, the polymerisation of actin and the acquisition by cancer cells of a spindle-shaped elongated morphology^50^. Altogether, these results suggest a critical role for Na_V_1.5 in the so-called “mesenchymal invasion”, in which cancer cells having a mesenchymal phenotype invade tissues thanks to their proteolytic capacity^52^. However, the participation of Na_V_ channels in the EMT is still elusive.

This study was aimed to elucidate the role of Na_V_1.5 in the EMT and its potential regulation by SIK1. Here, we show that Na_V_1.5 expression promotes EMT in breast cancer cells and is upregulated by TGF-β1. Furthermore, knocking down SIK1 expression induces Na_V_1.5 expression and is correlated with the increase of cancer cell invasiveness.

## Results

### Na_V_1.5 activity in breast cancer cells promotes the acquisition of a mesenchymal phenotype and invasive capacities

**Figure 1:**
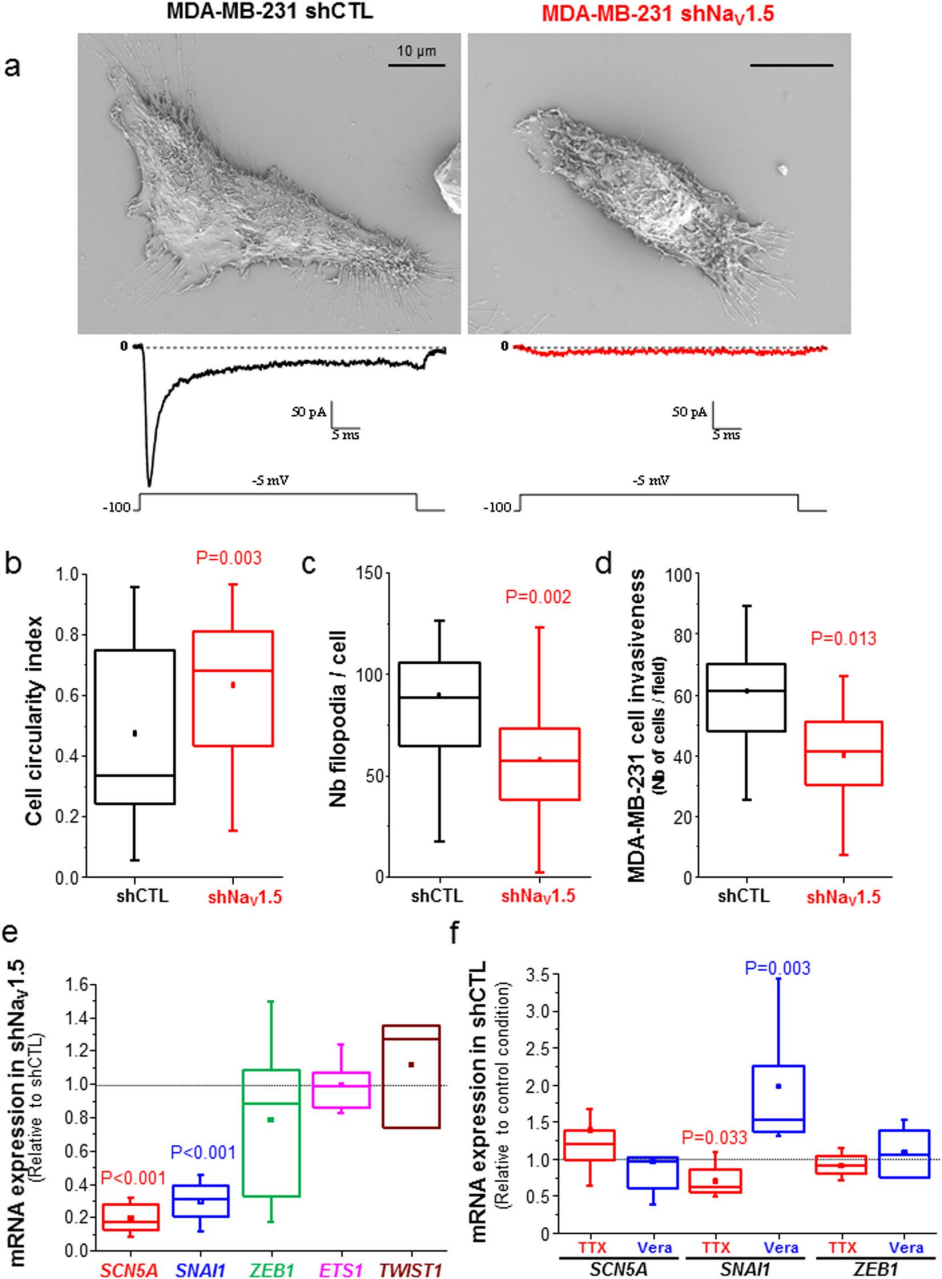
*SCN5A*/Na_V_1.5 expression and activity promotes the acquisition of a mesenchymal phenotype in MDA-MB-231 breast cancer cells. (**A**), Representative scanning electron microscopy micrographs performed from MDA-MB-231 shCTL and shNa_V_1.5 cells. Scale bar, 10 µm. As shown as an illustration underneath the micrographs, MDA-MB-231 shCTL cells (left panel) show fast Na_V_1.5-related inward current, with a small persistent component, when recorded with a voltage step from −100 mV to −5 mV, while MDA-MB-231 shNa_V_1.5 cells (right panel), knocked-down for the expression of the *SCN5A* gene, do not. **(B**) A cell circularity index was calculated from SEM micrographs taken from shCTL and shNa_V_1.5 cells (n = 40 and 67 cells per condition, respectively). P = 0.003 (Mann-Whitney rank sum test). **(C**) Number of filipods per cell counted from SEM micrographs taken from shCTL and shNa_V_1.5 (n = 24 and 29 cells per condition, respectively). P = 0.002 (Mann-Whitney rank sum test). **(D**) Summary of cancer cell invasiveness results from 3 independent experiments, for MDA-MB-231 shCTL and shNa_V_1.5 cells. Results indicate the total number of cells counted per field taken at the x20 objective. Five fields were counted for each individual Matrigel-coated invasion insert, n = 15 inserts (5 independent experiments). P = 0.0013 (Mann-Whitney rank sum test). **(E**) mRNA expression levels of *SCN5A*, *SNAI1*, *ZEB1, ETS1 and TWIST1* genes assessed by RT-qPCR in MDA-MB-231 shNa_V_1.5 cells and expressed as ratios to expression levels in MDA-MB-231 shCTL cells (n = 3–8 separate experiments). *SCN5A* and *SNAI1* were significantly lower in shNa_V_1.5 compared to shCTL cells (p < 0.001, Mann-Whitney rank sum test). **(F**) mRNA expression levels of *SCN5A*, *SNAI1* and *ZEB1* genes assessed by RT-qPCR in MDA-MB-231 shCTL cells treated with either tetrodotoxin (TTX, 30 µM) or veratridine (Vera, 50 µM) and expressed as ratios to control condition (vehicle). (n = 5–8 separate experiments). TTX treatment significantly reduced the expression level of *SNAI1* gene (P = 0.033, Mann-Whitney rank sum test) while Vera increased it (P = 0.003, Mann-Whitney rank sum test).

**Figure 2:**
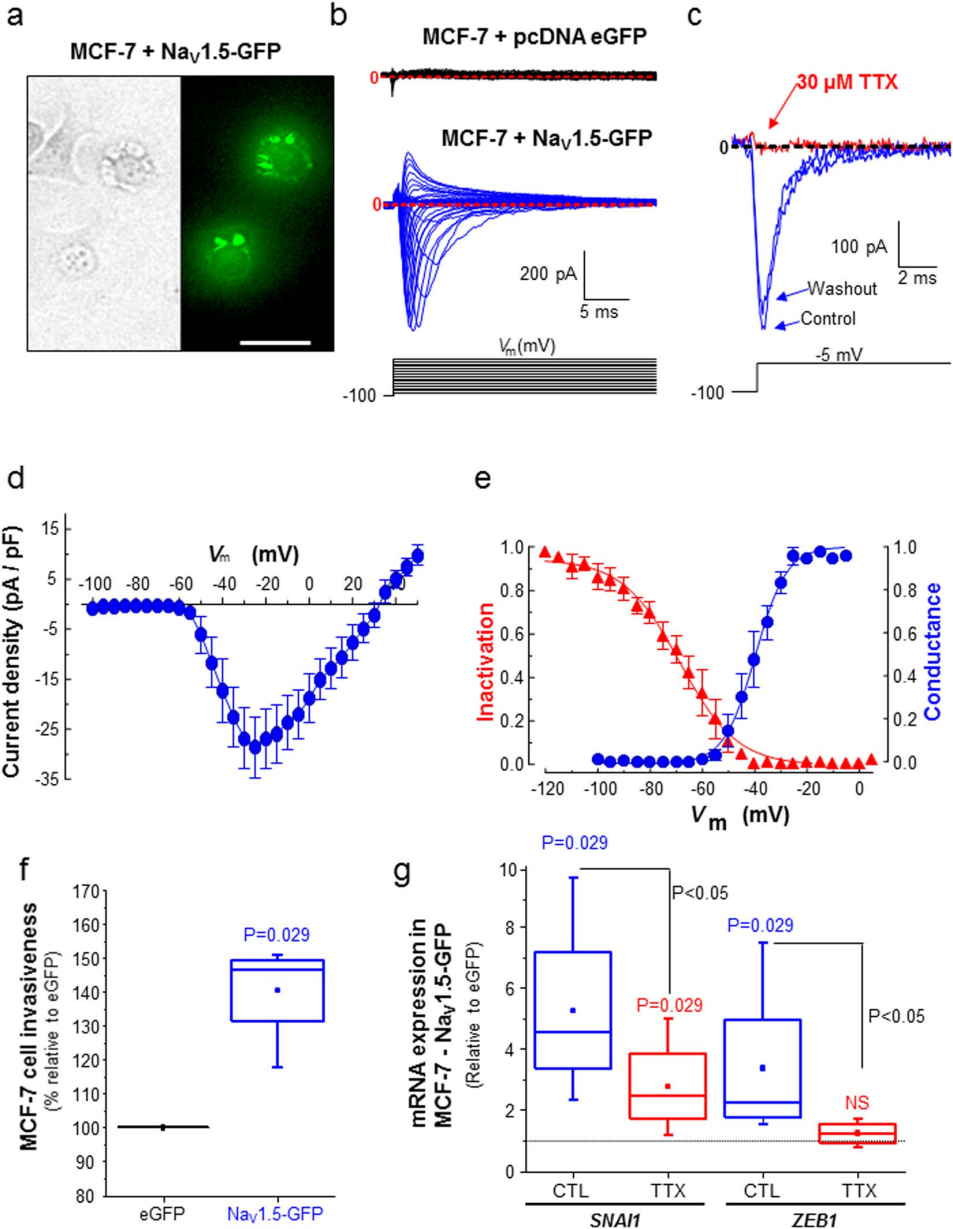
*SCN5A*/Na_V_1.5 heterologous expression in poorly aggressive MCF-7 cells increases invasive phenotype. **(A**) Representative micrographs showing MCF-7 cells transfected with Na_V_1.5-GFP in phase contrast (left) and in epifluorescence (right), 24 h after transfection. Scale bar, 20 µm. **(B**) Representative whole-cell current recordings obtained in MCF-7 cells transfected with either eGFP pcDNA3.1 (top recordings, black traces), or from MCF-7 cells transfected with NaV1.5-GFP pcDNA3.1 (bottom, blue traces), in response to depolarizing 30-ms pulses from −95 to +60 mV in 5-mV steps applied every 2 s from a holding potential of ─100 mV. Red dotted lines indicate baseline levels (zero current). **(C**) Representative whole-cell voltage-gated sodium currents recorded at −5 mV from a holding potential of −100 mV in absence (Control, PSS solution), presence of TTX (30 µM) and after washing out the toxin with PSS (Washout). **(D**) Current density-voltage (I-V) relationship for Na_V_1.5-GFP channels heterologously expressed in MCF-7 cells (n = 7). Peak sodium currents were averaged and plotted as a function of the depolarizing potential (Vm). **(E**) Conductance-voltage (blue circles) and steady-state inactivation-voltage (red triangles) relationships of peak Na^+^ currents recorded in MCF-7 cells heterologously expressing Na_V_1.5-GFP channels. Smooth lines are fits to Boltzmann functions and V_1/2_ values were calculated for each parameter. V_1/2_-activation: −39.5 ± 0.6 mV and V1/2-inactivation: −68.6 ± 1.2 mV. Data were obtained from five cells, and are presented as mean ± sem. **(F**) Invasiveness of MCF-7 cell transfected with Na_V_1.5-GFP expressed as a ratio to expression levels in cells transfected with eGFP. Data were obtained from 4 independent experiments, with 3 inserts per condition (P = 0.029, Mann-Whitney rank sum test). **(G**) mRNA expression levels of *SNAI1* and *ZEB1* genes assessed by RT-qPCR in MCF-7 cells transfected with Na_V_1.5-GFP and treated with tetrodotoxin (TTX, 30 µM) or not (CTL, vehicle) and expressed as ratios to expression levels in MCF-7 transfected with eGFP (n = 4 separate experiments, Mann-Whitney rank sum test). NS stands for “no statistical difference”.

Highly aggressive, triple-negative, MDA-MB-231 human breast cancer cells have been shown to be very invasive both *in vitro* and *in vivo*^43–45,53^. These cells, which endogenously express the *SCN5A* gene and display Na_V_1.5-dependent fast inward sodium currents^41,47^, show a typical spindle-shaped mesenchymal phenotype and multiple filopodia, as observed in scanning electron microscopy (Fig. 1a, left). However, when we stably knocked-down the expression of *SCN5A*, using a specific small-hairpin RNA (shNa_V_1.5 cells), thus leading to the absence of plasma membrane Na_V_1.5 currents, we noticed a dramatic change in the morphology of cancer cell that appeared to be less elongated (Fig. 1a, right). Indeed, we measured a significant increase of the cell circularity index in shNa_V_1.5 cells (median = 0.335, n = 40) compared to MDA-MB-231 cells expressing a null-target shRNA (shCTL cells, median = 0.679, n = 67, p = 0.003) (Fig. 1b), as well as a reduction in the number of filopodia per cell (median = 57 filopodia/shNa_V_1.5 cell, n = 39 *versus* 88.5 filopodia/shCTL cell, n = 24, p = 0.002) (Fig. 1c). Furthermore, the loss of *SCN5A* expression resulted in a 33%-reduction of MDA-MB-231 cell invasiveness through matrigel-coated inserts (Fig. 1d, p = 0.013). These results are in line with previously published data using tetrodotoxin (TTX) to block Na_V_1.5 activity, and demonstrating a rapid loss of mesenchymal phenotype^50^. Therefore, we assessed the expression level of EMT-inducing transcription factors in shNa_V_1.5 compared to more invasive shCTL breast cancer cells, and identified that *SNAI1* expression was specifically and significantly reduced by 69.4% (p < 0.001), while the expression of other EMT-promoting transcription factors ZEB1, *ETS1* and *TWIST1* was not affected (Fig. 1e). Correlatively, the pharmacological inhibition of Na_V_1.5 using TTX (30 µM) in Nav1.5-expressing shCTL cells reduced *SNAI1* expression by 39% (p = 0.033), while its activation using veratridine (50 µM) had opposite effects and induced its expression by 52% (P = 0.003). TTX and veratridine treatments had no effect either on *SCN5A* or on *ZEB1* expression (Fig. 1f). These results support a role for both Na_V_1.5 expression and activity in maintaining a mesenchymal phenotype in aggressive cancer cells. We then questioned whether the experimental heterologous overexpression of Na_V_1.5 in weakly invasive and epithelial-type breast cancer cells, which do not express the protein endogenously, could promote EMT and increase invasiveness. Therefore, we transfected MCF-7 cells, known to express Na_V_1.5 mRNA and proteins that remain intracellular and do not show functionality at the plasma membrane^42,49^, with either a plasmid (pcDNA3.1- hNa_V_1.5-GFP) encoding for human Na_V_1.5 tagged with eGFP at its C-terminus or with a plasmid (pcDNA3.1-eGFP) encoding for only eGFP. Even though the transfection efficacy was quite low with both plasmid types (<30%), an increased amount of Na_V_1.5 proteins was measured in cells transfected with pcDNA-Na_V_1.5-GFP showing a green fluorescence emission (Fig. 2a). The epifluorescence imaging showed a strong GFP signal in the perinuclear area, which most probably corresponded to intracellular organelles involved into the membrane protein translation, maturation and addressing (i.e. endoplasmic reticulum, Golgi)^54^, as well as a plasma membrane labelling. The functionality of Na_V_1.5 at the plasma membrane of MCF-7 cells transfected with pcDNA-Na_V_1.5-GFP was verified by the recording of voltage-gated transient currents that were absent in cells transfected with pcDNA-eGFP (Fig. 2b). These transient currents were inhibited by 30 µM TTX confirming the activity of Na_V_ channels (Fig. 2c). Na_V_1.5-mediated sodium currents recorded from transfected MCF-7 show typical current-voltage relationships, with a threshold of activation at −60 mV and a maximal peak current of –23.6 ± 5.4 pA/pF (mean ± sem, n = 7) at −10 mV (Fig. 2d). Fitting activation-voltage and inactivation-voltage relationships of sodium currents gave V_1/2_-activation and V_1/2_-inactivation voltages of −39.5 ± 0.6 mV and −68.6 ± 1.2 mV, respectively (Fig. 2e). A window of voltage was present between −60 and −35 mV and the mean membrane potential was −36.9 ± 6.9 mV (n = 7 cells), suggesting that a window current could allow a persistent entry of sodium into the cells. This heterologous expression of Na_V_1.5 in MCF-7 cells, albeit with a poor efficacy, resulted in a significant increase of invasiveness by 46.6% (p = 0.029) (Fig. 2f), as well as the increase of both *SNAI1* (x4.5) and *ZEB1* (x2.2) expression, that were prevented by the treatment with TTX (Fig. 2g).

### The EMT inducer TGF-β1 stimulates SCN5A expression and Na_V_1.5-mediated invasiveness

**Figure 3:**
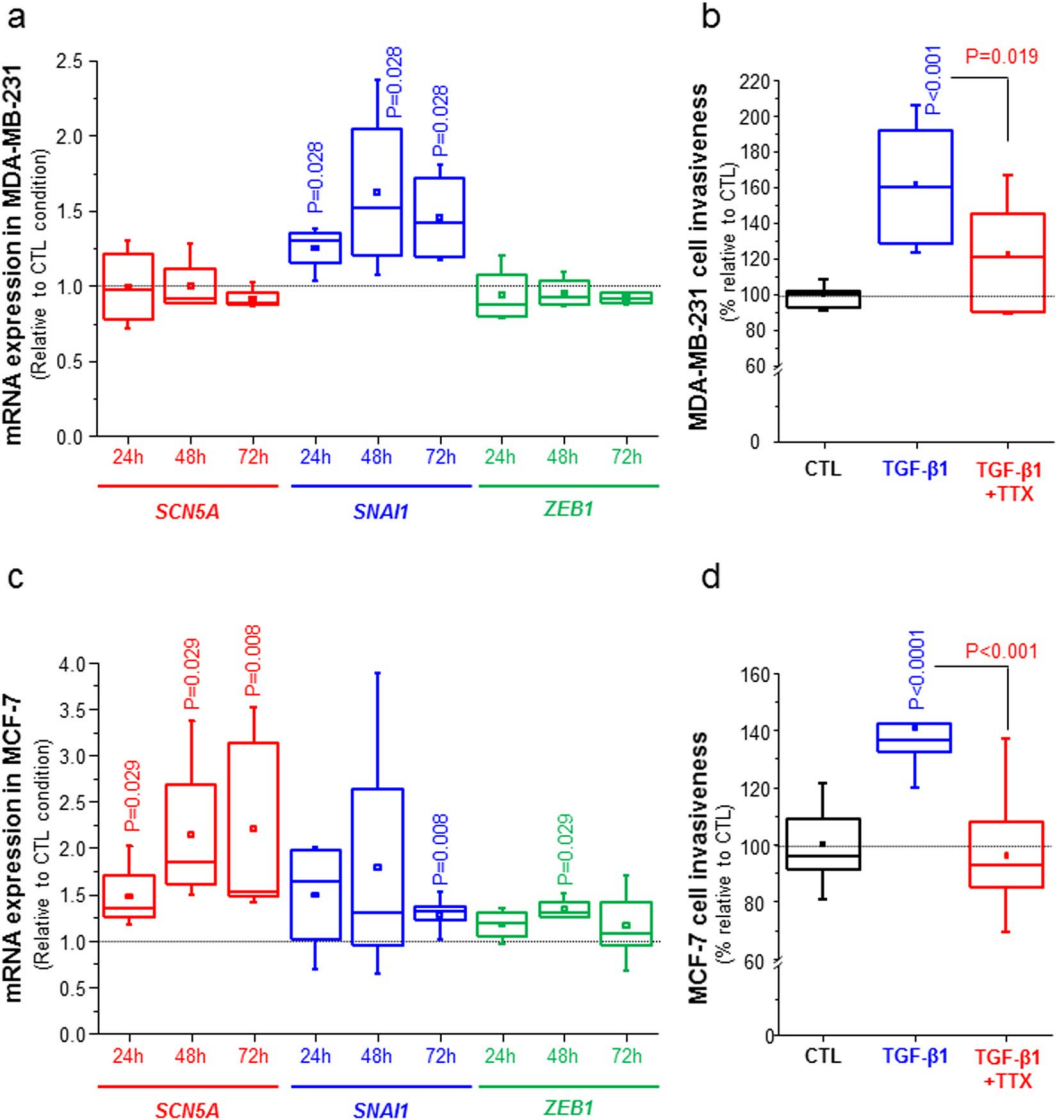
TGFβ1 treatment enhances *SCN5A* expression and invasive phenotype. (**A**) Effect of TGF-β1 (5 ng/mL) on the expression of *SCN5A*, *SNAI1* and *ZEB1* genes in MDA-MB-231 cells, 24 h, 48 h or 72 h after the treatment. Results were obtained by RT-qPCR from 4 independent experiments and expressed as a ratio to the control condition (vehicle). P values indicate significant differences compared with the control condition at the same time point (Mann-Whitney rank sum test), otherwise there is no statistical difference. (**B**) Effect of TGF-β1 (5 ng/mL for 24 h) on the invasiveness of MDA-MB-231 cells. Data were obtained from 3 independent paired experiments, with 3 inserts per condition, in the absence or presence of 30 µM TTX. p < 0.0001, when comparing TGF-β1 to CTL, and p < 0.001 when comparing TGF-β1 to TGF-β1 + TTX (Mann-Whitney rank sum test). (**C**) Effect of TGF-β1 (5 ng/mL) on the expression of *SCN5A*, *SNAI1* and *ZEB1* genes in MCF-7 cells, 24 h, 48 h or 72 h after the treatment. Results were obtained by RT-qPCR from 4 independent experiments and expressed as a ratio to the control condition (vehicle). P values indicate significant differences compared with the control condition at the same time point (Mann-Whitney rank sum test), otherwise there is no statistical difference. (**E**) Effect of TGF-β1 (5 ng/mL for 24 h) on the invasiveness MCF-7 cells, in presence or absence of 30 µM TTX. Data were obtained from 3 independent paired experiments, with 3 inserts per condition. p < 0.001, when comparing TGF-β1 to CTL, and P = 0.019 when comparing TGF-β1 to TGF-β1 + TTX (Mann-Whitney rank sum test).

TGF-β1 is a well-known signal coming from the mesenchymal stroma and inflammatory cells of the tumour, acting as a primary inducer of EMT and metastases development^55^, and we questioned whether it could potentiate *SCN5A* expression and mesenchymal phenotype. MDA-MB-231 cells treated with TGF-β1 (5 ng/mL) demonstrated an increase of *SNAI1* expression, measured in qPCR, at 24 h (+25%, p = 0.028), at 48 h (+87%, p = 0.028) and at 72 h (+45%, p = 0.028) compared to control condition. However, TGF-β1 but did not induce *SCN5A* which is endogenously expressed in MDA-MB-231, nor *ZEB1* (Fig. 3a). TGF-β1 significantly increased MDA-MB-231 invasiveness by 61%, and this effect was prevented by the use of TTX (Fig. 3b).

Weakly invasive MCF-7 cells treated with TGF-β1 also displayed a +31.1% induction of *SNAI1* at 72 h and a +30.0% induction of *ZEB1* at 48 h. For these two genes, a tendency for an induction of expression by TGF-β1 was observed for the two other times considered (Fig. 3c). This treatment also induced the expression of *SCN5A* gene by 35.2% at 24 h (p = 0.029), 85.3% at 48 h (p = 0.029) and 53.3% at 72 h (p = 0.008) (Fig. 3c). This treatment resulted in a 40.8% increase of MCF-7 invasion (p < 0.001), which was prevented by the use of TTX (Fig. 3d). These results indicate that EMT-inducing conditions, such as the stimulation of cancer cells with TGF-β1, could promote *SCN5A* expression and Na_V_1.5-dependent increase in invasive capacities.

### Loss of SIK1 expression correlates with breast cancer progression, stimulates SCN5A expression and Na_V_1.5-mediated invasiveness

**Figure 4:**
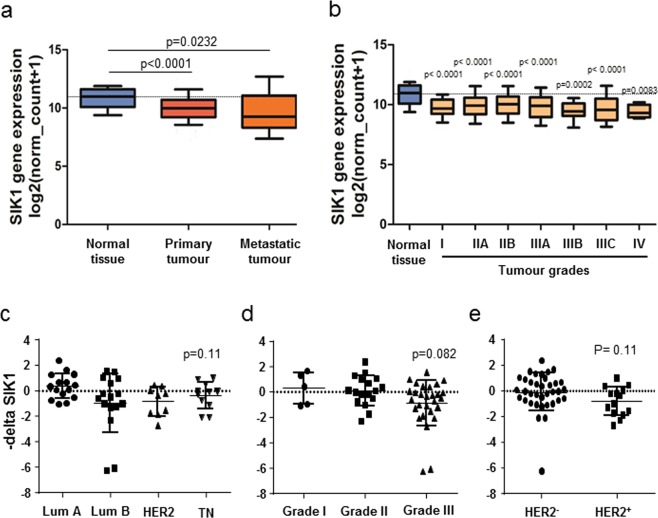
The Salt-inducible Kinase type 1 (SIK1) is downregulated in breast cancer. (**A**) The expression level of *SIK1* in normal breast (normal tissue, n = 114), primary breast tumours (n = 1097) and metastatic tumours (n = 7) is analysed from datasets coming from the “The Cancer Genome Atlas” (http://cancergenome.nih.gov) from the US National Cancer Institute. For each array, data were log2-transformed and centered to the median. P values indicate significant differences (Mann-Whitney rank sum test) as compared to normal tissue. (**B**) The expression level of *SIK1* in normal breast (normal tissue, n = 114) and in different breast tumour grades: I (n = 125), IIA (n = 243), IIB (n = 115), IIIA (n = 85), IIIB (n = 10), IIIC (n = 31), IV (n = 4). P values indicate significant differences (Mann-Whitney rank sum test) as compared to normal tissue. (**C**–**E**) Expression level of *SIK1* assessed by RT-qPCR from breast cancer biopsies obtained from the University Hospital of Tours and expressed as –deltaCt using *HPRT1* as a reference gene, (**C**) depending on breast cancer subtypes: luminal A (Lum A, n = 15), luminal B (Lum B, n = 16), HER2-enriched (HER2, n = 10) and triple-negative breast tumour (TN, n = 11). (**D**) depending on tumour grade: I (n = 5), II (n = 17) and III (n = 28). (**E**) depending on the expression of HER2 negative (n = 35) or positive (n = 14).

SIK1 has been identified as an important protagonist controlling sodium homeostasis^15,16^ and was recently proposed as acting as a tumour-suppressor^19^. An *in silico* study confirmed that *SIK1* expression was reduced in breast primary and metastatic breast tumours, as compared with normal tissues (Fig. 4A). This lower expression of *SIK1* gene in breast tumours, compared to normal mammary tissues, was observed at all tumour stages (Fig. 4b). Breast tumour biopsies obtained from the Tours University Hospital were analysed to assess *SIK1* expression in the different breast tumour subtypes. While the statistical significance was not reached, a tendency was observed for a lower expression in triple-negative tumours, compared to luminal A, luminal B, or HER2 (Fig. 4c), a lower expression in grade III, compared to grades I and II (Fig. 4d), and a lower expression in HER2+ compared to pooled HER2- tumours (Fig. 4e).

**Figure 5:**
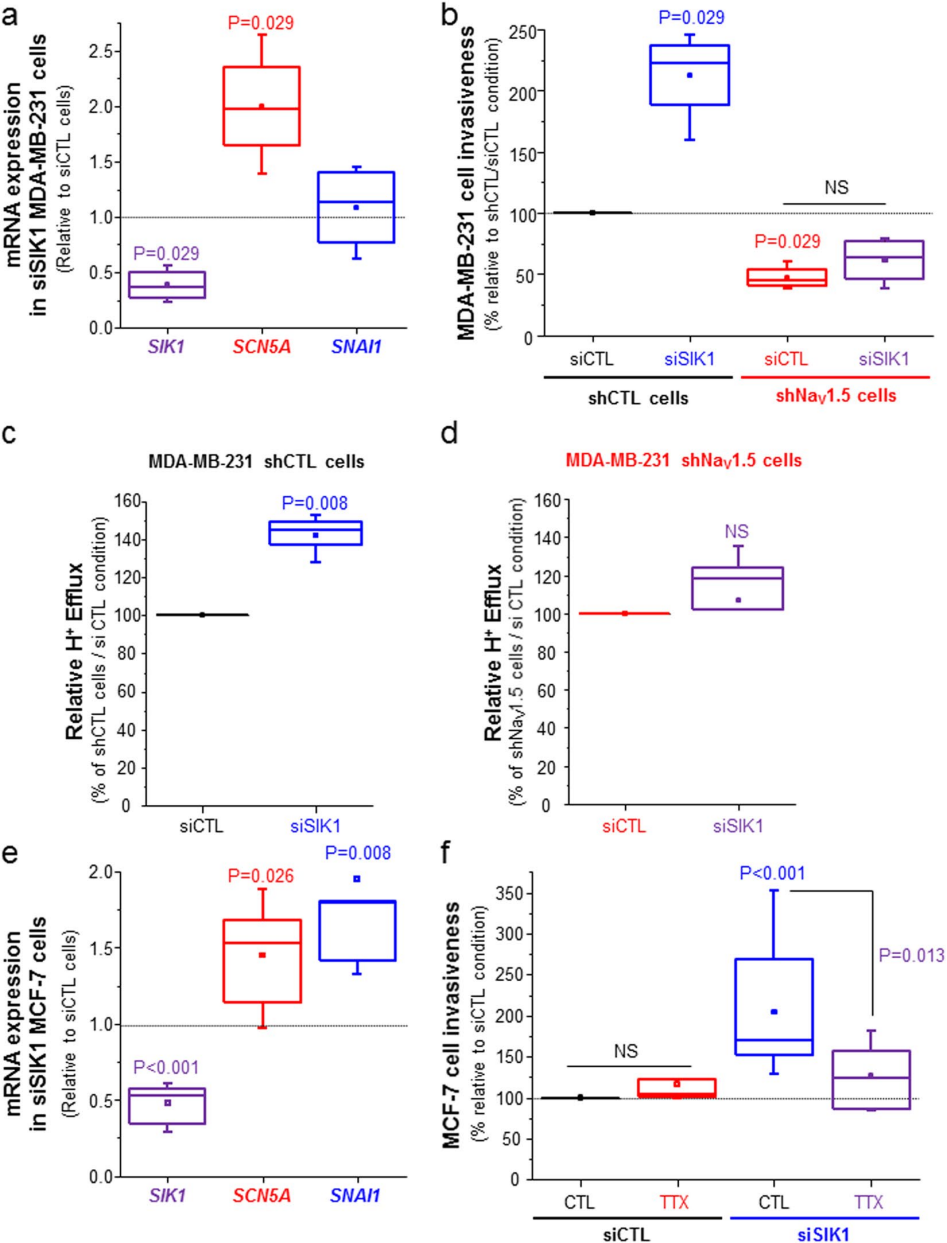
Knocking down SIK1 expression increases Na_V_1.5-dependent cancer cell invasiveness. (**A**) Effect of silencing *SIK1* (siSIK1) in MDA-MB-231 cells on the mRNA expression of *SIK1*, *SCN5A* and *SNAI1* genes. Results were obtained from 4 independent RT-qPCR experiments, 24 h after siRNA transfection, and are expressed relatively to the results obtained when transfecting control irrelevant siRNA (siCTL). P values indicate significant differences (Mann-Whitney rank sum test) as compared to siCTL condition. **(B**) Effect of silencing *SIK1* (siSIK1) on the invasiveness of MDA-MB-231 shCTL and shNa_V_1.5 cells. Results are coming from 4 independent experiments and are expressed relatively to the effect of the siCTL condition in shCTL cells. P values indicate significant differences (Mann-Whitney rank sum test) as compared to siCTL condition in shCTL cells. NS stands for “no statistical difference” between siCTL and siSIK1 conditions in shNa_V_1.5 cells. **(C**) Relative H^+^ efflux measurements induced by reintroduction of 130 mM NaCl in MDA-MB-231 shCTL cells transfected with either siCTL or siSIK1, after a NH_4_Cl wash pulse-induced intracellular acidification in a Na^+^-free solution. Results are coming from 5 independent experiments and are expressed relatively to the efflux measured in the siCTL condition. P value indicates a significant difference (Mann-Whitney rank sum test) when compared to the siCTL condition. **(D**) Relative H+ efflux measurements induced by reintroduction of 130 mM NaCl in MDA-MB-231 shNa_V_1.5 cells transfected with either siCTL or siSIK1, as in C. Results are coming from 5 independent experiments and are expressed relatively to the efflux measured in the siCTL condition. NS indicates “no statistical difference” when compared to the siCTL condition. **(E**) Effect of silencing SIK1 (siSIK1) in MCF-7 cells on the mRNA expression of *SIK1*, *SCN5A* and *SNAI1* genes. Results were obtained from 7 independent RT-qPCR experiments, 24 h after siRNA transfection, and are expressed relatively to the results obtained when transfecting control irrelevant siRNA (siCTL). P values indicate significant differences (Mann-Whitney rank sum test) as compared to the siCTL condition. **(F**) Effect of silencing SIK1 (siSIK1) on the invasiveness of MCF-7 cells, in the presence of 30 µM tetrodotoxin (TTX) or not (CTL, vehicle). Results are coming from 7 independent experiments and are expressed relatively to the effect of the CTL condition in siCTL cells. P values indicate significant differences (Mann-Whitney rank sum test). NS stands for “no statistical difference” between CTL and TTX conditions in siCTL cells.

The use of a specific siRNA to knock down the expression of *SIK1* in MDA-MB-231 cells (inhibition by 63.5%, p = 0.029) increased the expression of *SCN5A* by +97.5% (p = 0.029) with no effect on *SNAI1*, 24 h after transfection (Fig. 5a). This resulted in a +122.2% increase of invasiveness in shCTL cells (p = 0.029). In comparison, knocking-down the expression of *SIK1* in shNa_V_1.5 cells, which present a lower invasive capacity as compared to shCTL cells, had no stimulating effect on invasiveness (Fig. 5b). Because Na_V_1.5 was demonstrated to promote breast cancer cell invasiveness through a promotion of extracellular acidification involving NHE1 allosteric activation^49,50^, we monitored the H^+^ efflux at the introduction of NaCl in MDA-MB-231 cells previously acidified (NH_4_Cl pulse-wash protocol in sodium-free medium). We noticed that silencing *SIK1* promoted H^+^ efflux in shCTL by +44.5% (Fig. 5c), but not in shNa_V_1.5 cells (Fig. 5d). In shCTL and shNa_V_1.5 cells, knocking-down *SIK1* had no effect on *NHE1* mRNA expression monitored by qPCR (data not shown). Correlatively, knocking-down the expression of *SIK1* in MCF-7 cells also induced the expression of *SCN5A* by +52.8% (p = 0.026), of *SNAI1* by +79.6% (p = 0.08) (Fig. 5e), and invasiveness by +70.1% (p < 0.01) (Fig. 5f). This increase in MCF-7 cell invasiveness was prevented by the use of 30 µM TTX (Fig. 5f).

Taken together, these results indicate that the loss of *SIK1* participate in promoting breast cancer cell invasiveness through the induction of *SCN5A* expression and Na_V_1.5 activity.

## Discussion

Increasing evidence indicates that pore-forming α^32,56^ and auxiliary β subunits^53,57–60^ of voltage-gated sodium channels are key contributors of oncogenic properties and metastatic progression. Particularly, pore-forming Na_V_ α have been demonstrated to be overexpressed in a wide range of biopsies and cancer cells originating from different types of carcinomas, including breast cancer^42,46^, prostate cancer^39,61^, lung cancer^35,62^, cervical cancer^36,38,40^, ovarian cancer^63^, gastric cancer^64^ and colon cancer^37,65^. In all these cancer types, different isoforms of pore-forming Na_V_ are expressed at the mRNA level, probably also at the protein level, but generally only one is functional at the plasma membrane of cancer cells giving rise to recordable sodium currents, with the only exception so far of the non-small-cell cancer cell line Calu-1 in which several Na_V_α might simultaneously be functional^35^. In all cases, Na_V_ expression has been shown to increase the invasive potency and the extracellular matrix degradation capacity. Several studies identified that functional Na_V_ expressed at the plasma membrane of breast, colon and cervical cancer cells were neonatal splice variants^40,42,66–68^, and not the adult splice variant isoform that might be restricted to non-cancer excitable cells. Whether this is the case for other types of cancers still has to be investigated.

The use of pharmacological inhibitors such as TTX^47^, the anticonvulsant phenytoin^45^, the antianginal ranolazine^44,69^, the local anaesthetics lidocaine or ropivacaine^67^, or even newly developed small Na_V_ inhibitory molecules^48^, has been shown to reduce cell invasiveness, albeit not completely abrogating it, and to modify cancer cell morphology to a less aggressive phenotype. By comparison, the use of pharmacological Na_V_ inhibitors in weakly invasive cancer cells not showing any plasma membrane sodium current, yet expressing Na_V_ mRNA and protein, such as MCF-7 breast^46,49^ or A-549 non-small cell cancer cells^35^ had no effect on cell invasiveness. Taken together all of these results strongly suggest that the functionality of Na_V_ at the plasma membrane, i.e. the sodium influx, independently on Na_V_ molecular nature, is critical for the gain of aggressiveness in cancer cells. However, this does not exclude the possibility that Na_V_ expression in intracellular compartments might also be involved in cancer cell migration, invasion, endocytic recycling or phagocytic activity, as is the case for microglial and immune cells^26,29^. Nevertheless, in almost all of these previously published studies, researchers inhibited the expression or activity of Na_V_ in aggressive, endogenously Na_V_-expressing cancer cells. In this study, we demonstrated that the heterologous expression of Na_V_ in weakly invasive cancer cells is sufficient to increase cell invasiveness. Furthermore, the adult splice variant of the channel that we heterologously overexpressed seems to promote cancer cell invasiveness similarly to the neonatal one, further supporting the idea that NaV activity and not the NaV protein *per se*, as a structural molecule, is important to cancer progression. These results are in line with recently published data obtained with the overexpression of the adult Na_V_1.6 isoform in C33A cervical cancer cells^40^. The reason why some Na_V_ isoforms are specifically expressed and correlated to some specific cancers, Na_V_1.5 in breast and colon cancers, Na_V_1.6 in cervical cancer, Na_V_1.7 in prostate cancer, remains to be determined.

In MDA-MB-231 breast cancer cells, Na_V_1.5 activity was shown to promote invadopodial activity and the proteolytic degradation of the extracellular matrix^47,49,50^. Furthermore, Na_V_1.5 activity was shown to sustain the polymerisation of actin, the generation of F-actin stress fibres and the acquisition by cancer cells of a fibroblast-like morphology^50^. These results suggested a role for Na_V_1.5 in “mesenchymal invasion”. However, the participation of Na_V_ activity in the epithelial-to-mesenchymal dedifferentiation was unclear. In this study, we demonstrated for the first time that both the expression and the activity of Na_V_1.5 sustain the expression of the EMT-promoting transcription factor *SNAI1*, the acquisition of a mesenchymal phenotype and of enhanced invasive capacities. In weakly invasive and epithelial-phenotype MCF-7 cells, the overexpression and activity of Na_V_1.5 increased the expression of *SNAI1* but also induced *ZEB1*. Our results using both pharmacological tools and shRNA in cells endogenously expressing Na_V_1.5, and in cells heterologously expressing Na_V_1.5, are in apparent contradiction with data published by Nelson and collaborators^45^. In this previously published study, MDA-MB-231 cells stably knocked-down for the expression of Na_V_1.5 (shRNA transduction) did not show any significant reduction in the protein expression from *SNAI1*, although a tendency for a small reduction could be observed on the western blot shown, and no effect on vimentin or SLUG expression. Only a reduction in CD44 was identified. A possible explanation for this discrepancy could be the selection, in their study, of a specific clone expressing the transduced shRNA.

In weakly aggressive MCF-7 breast cancer cell line, TGF-β1 stimulation promoted Na_V_1.5 expression, EMT transcription factors expression and mesenchymal invasion. Considering the fact that the treatment with TGF-β1 increased *SCN5A* expression, and that TGF-β1-induced increase in MCF7 cancer cell invasiveness was prevented by the use TTX, it is most likely that Na_V_1.5 channels expressed and functional (giving rise to sodium currents) at the plasma membrane of cancer cells are responsible for this effect. Indeed, electrophysiological experiments performed in MCF7 treated with TGF-β1 (for 72 h) showed in several, but not all cells, the apparition of transient inward currents (Suppl. Fig. 1) which are absent in non-treated cells (Fig. 2b) and could correspond Na_V_1.5-mediated sodium currents. However, we cannot exclude the possibility that intracellular Na_V_1.5 channels might also be involved in the regulation of cancer cell invasiveness.

It is of interest to compare these results, obtained in human breast cancer cells, to data acquired with cardiac cells expressing Na_V_1.5. While TGF-β1 might be important for the embryogenic development of the heart, its overexpression has also been associated with cardiac remodelling and heart diseases^70^. Indeed, a regulation of cardiac Na_V_1.5 by TGF-β1 seems to be associated with cardiac fibrosis and conduction troubles. In neonatal rat cardiac myocytes, TGF-β1 treatment reduced Na_V_1.5 activity with a 30%-decreased peak Na^+^ current, which could be attributed to a reduced protein expression of the channel, combined with a leftward shift of the inactivation-voltage relationship^71^. Scn5a haplo-insufficient mice (Scn5a+/-), thus showing a loss-of-function of Na_V_1.5, are characterized by impaired sinoatrial node automatic activity and slowed sinoatrial conduction. Interestingly, it is also associated with increased collagen and fibroblast levels, high levels of TGF-β1 and vimentin. Loss of Na_V_1.5 was associated with a TGF-β1-mediated cardiac fibrosis^72^. More recently, Derangeon and collaborators demonstrated, in a model of progressive cardiac conduction disease, that the ventricular fibrosis that develops with aging in Scn5a+/- mice is secondary to the activation of the TGF-β1 signalling pathway^73^. Cardiac fibrosis could result from an increased collagen expression and maturation from cardiac fibroblasts, or from a decrease in the collagen fibres turnover, which is normally regulated by proteolytic enzymes. It is of particular interest to notice that a loss-of-function of Na_V_1.5 in cardiac cells promotes collagen deposit and fibrosis, while its gain of function in cancer cells induces extracellular matrix degradation and invasion. The fact that in one case TGF-β1 reduces Na_V_1.5 expression and activity in cardiac cells, but increases them in cancer cells might be representative of a complex feedback regulation that might be lost during carcinogenesis.

The secondary objective of this study was to determine whether Na_V_ expression in cancer cells could be a part of a general dysregulation of the SIK signalling pathway, known to control sodium homeostasis. A reduced expression of *SIK1* has been identified in breast cancer and has been associated with metastatic progression and with a poor outcome^17,18^. In other cancer types, the loss of *SIK1* expression promoted the expression of EMT markers, as well as invasive capacities both *in vitro* and *in vivo*^20,21^. Indeed, SIK proteins have been demonstrated to participate in gene regulation^74^.

In the current study, we confirmed a reduction of SIK1 expression in breast tumours, and further identified that reducing SIK1 expression in breast cancer cells induced *SCN5A* expression and Na_V_1.5-dependent invasiveness. The loss of SIK1 expression in epithelial-like phenotype MCF-7 cells promoted the expression of *SNAI1*, but did not significantly modulate its expression in MDA-MB-231 cancer cells already largely engaged in a mesenchymal phenotype.

Taken together, these results suggest that loss of SIK1 in cancer cells might be a critical step in tumour progression by inducing Na_V_1.5 expression and activity, triggering the epithelial-to-mesenchymal transition, and the acquisition of pro-metastatic capacities that might be associated with a complete dysregulation of Na^+^ homeostasis in the cancer.

## Methods

### *In Silico* RNA expression

The expression level of *SIK1* gene in breast cancer tissues was performed using data available from “The Cancer Genome Atlas” for breast invasive carcinoma (TCGA BRCA) by using Cancer Genomics Browser (http://xena.ucsc.edu/).

### Chemicals and drugs

Tetrodotoxin was purchased from Latoxan (France). All other drugs and chemicals were purchased from Sigma-Aldrich (France).

### Breast tumour biopsies

Breast cancer biopsies, coming from surgical interventions performed by the gynaecological surgery department of the University-Hospital of Tours, were frozen in liquid nitrogen and conserved at the tumour collection (N°DC2008-308). All experiments and methods were performed in accordance with relevant guidelines and regulations, and approved by the “Comité de Protection des Personnes” (CPP) of Tours hospital. Informed consent was obtained from all subjects.

### Cell lines and culture

Cell lines were purchased from the American Type Culture Collection (LGC Promochem, France). Cells were grown at 37 °C in 5% CO_2_ incubator, in a humidified atmosphere. MCF-7 and MDA-MB-231 breast cancer cells were cultured in DMEM supplemented with 5% foetal calf serum (FCS). MDA-MB-231 shCTL and shNa_V_1.5 breast cancer cells were generated as previously described^44,53^, from MDA-MB-231 cells stably expressing the luciferase gene. Briefly these two cell lines were obtained by transduction with a lentiviral vector encoding a short hairpin RNA (shRNA) specifically targeting human *SCN5A* transcripts (shNa_V_1.5 cell line) or a null-target shRNA (shCTL cell line). The sequence encoding sh*SCN5A*, inhibiting the expression of Na_V_1.5 protein, was obtained by DNA polymerase fill-in of two partially complementary primers: 5′-GGATCCCCAAGGCACAAGTGCGTGCGCAATTCAAGAGA-3′ and 5′-AAGCTTAAAAAAAGGCACAAGTGCGTGCGCAATCTCTTGAA-3′. We constructed a lentiviral vector expressing a null-target shRNA (pLenti-shCTL), using the following primers: 5′-GGATCCCCGCCGACCAATTCACGGCCGTTCAAGAGACG-3′ and 5′-AAGCTTAAAAAGCCGACCAATTCACGGCCGTCTCTTGAACG-3′.

Tests assessing for mycoplasma contamination were performed once a week, every week (Lonza, MycoAlert™ Mycoplasma Detection Kit).

### RNA extraction, reverse transcription, and real-time PCR

Total RNA extraction was performed (RNAgents® Total RNA Isolation System, Promega, France) and RNA yield and purity were determined by spectrophotometry. Only samples with an A260/A280 ratio above 1.6 were kept for reverse-transcription. To do so, RT kits Ready-to-go® You-prime First-Strand Beads (Amersham Biosciences, UK) and random hexamers pd(N)_6_ 5′-Phosphate (0.2 µg, Amersham Biosciences) were used. Samples were incubated at 37 °C for 60 min. Real time PCR experiments were performed as previously described^47^. Results obtained from cell lines are expressed as the relative gene expression using the comparative 2^−ΔΔCt^ method^75^ with *PPIA* as a reference gene. Results coming from breast cancer biopsies, for which there are no associated non-cancer tissues and no experimental conditions, were expressed as – ΔCt using *HPRT1* as a reference gene. Primers sequences can be found in Table 1.

**Table 1:** PCR primers sequences and expected amplicon size.

Gene	protein	Forward primers (5′ → 3′)	Reverse primers (5′ → 3′)	Expected size (bp)
*ETS1*	v-ets erythroblastosis virus E26 oncogene homolog 1	CTGCGCCCTGGGTAAAGA	CCCATAAGATGTCCCCAACAA	65
*HPRT1*	Hprt1	TTGCTGACCTGCTGGATTAC	TATGTCCCCTGTTGACTGGT	119
*PPIA*	Peptidylprolyl Isomerase A (PPIA), Cyclophilin A	ACCGCCGAGGAAAACCGTGTA	TGCTGTCTTTGGGACCTTGTCTGC	129
*SCN5A*	Na_V_1.5	CACGCGTTCACTTTCCTTC	CATCAGCCAGCTTCTTCACA	208
*SIK1*	Salt-Inducible Kinase 1 (SIK1)	TCCAGACCATCTTGGGGCAG	AAGGGGAAGGGGTTTTGTGTTG	86
*SLC9A1*	Sodium-proton Exchanger type 1 (NHE1)	TCTTCACCGTCTTTGTGCAG	AAGGTGGTCCAGGAACTGTG	125
*SNAI1*	Snai1	AATCCAGAGTTTACCTTCCAGCA	TCCCAGATGAGCATTGGCAG	110
*TWIST1*	Twist1	CGGGAGTCCGCAGTCTTA	GCTTGAGGGTCTGAATCTTG	161
*ZEB1*	Zeb1	TGCACTGAGTGTGGAAAAGC	TGGTGATGCTGAAAGAGACG	237

### Transfection of cells with plasmid and small interfering RNA

MDA-MB-231 and MCF-7 human breast cancer cells were transfected with 20 nM small interfering RNA (siRNA, Tebu-Bio, France) targeting the expression of *SIK1* (si*SIK1*, sc-91428) or scrambled siRNA (siCTL, siRNA-A sc-37007). SiRNA Transfections were performed with Lipofectamine RNAi max (Invitrogen, France). For some experiments, MCF-7 cells were transfected with a pcDNA3.1 plasmid encoding for human Na_V_1.5-GFP (generous gift of Dr. Pascale Guicheney, Inserm UMR_S 956, UPMC, Paris), or alternatively by a pcDNA3.1 plasmid encoding for enhanced green fluorescent protein (eGFP). Cells, at a 80–90% confluence state, were transfected with 1 µg cDNA using Lipofectamine 2000 (Invitrogen, France). Transfection efficiency was verified by qPCR using an iCycler^®^ system (BioRad, USA), and by western blotting.

### Electrophysiology

Whole-cell currents were recorded, as already described^46^, under the voltage-clamp mode of the patch-clamp technique, at room temperature, using an Axopatch 200B patch clamp amplifier (Axon Instrument, USA). Patch pipettes were pulled from borosilicate glass (TW150-3, World Precision Instruments, France) to a resistance of 3–5 MΩ using. Analogue signals were filtered at 5 kHz, and sampled at 10 kHz using a 1440 A Digidata converter. Cell capacitance and series resistance were electronically compensated by about 60%. The P/2 sub-pulse correction of cell leakage and capacitance was used to study Na^+^ current (I_Na_). Sodium currents were recorded by depolarizing the cells from a holding potential (HP) of –100 mV to a maximal test pulse of –30 mV for 30 ms every 500 ms. Sodium current-voltage (I_Na_-V) relationships were determined using the following protocol: the membrane was depolarized from a HP of –100 mV to potentials from –80 to +60 mV, with 5-mV increments, for 50 ms and at a frequency of 2 Hz. Availability-voltage relationships were obtained by applying 50 ms prepulses using the I_Na_-V curve procedure, followed by a depolarizing pulse to –5 mV for 50 ms. Currents were normalized to the amplitude of the test current without a prepulse. Currents amplitudes were normalized to cell capacitance and expressed as current density (pA/pF). The Physiological Saline Solution (PSS) had the following composition (in mM): NaCl 140, KCl 4, MgCl_2_ 1, CaCl_2_ 2, D-Glucose 11.1, and HEPES 10, adjusted to pH 7.4 with NaOH (1 M). The intrapipette solution had the following composition (in mM): KCl 130, NaCl 15, CaCl_2_ 0.37, MgCl_2_ 1, Mg-ATP 1, EGTA 1, HEPES 10, adjusted to pH 7.2 with KOH (1 M).

### Intracellular pH (pHi) measurements

Cells were incubated for 30 min at 37 °C in Hank’s medium containing 2 µM BCECF-AM (2′,7′-bis-(2-carboxyethyl)-5-(and-6)-carboxyfluorescein; excitation 503/440 nm; emission 530 nm), then washed twice with PSS. H^+^ efflux was measured after an NH_4_Cl pulse-wash intracellular acidification protocol in absence of external sodium, followed by 130 mM NaCl extracellular introduction allowing re-alkalinisation, as previously described^49,76^.

### Cell viability

Cancer cells were seeded in a 24-well plate at the density of 4 × 10^4^ cells per well. Media were changed every day, and after 5 days growing, the number of viable cells was assessed by the tetrazolium salt assay as previously described^47^ and normalised to the appropriate control condition.

### Cancer cell invasiveness assessment

Cancer cell invasiveness was using culture inserts with 8-µm pore size filters covered with Matrigel™ (Becton Dickinson, France), as already described^77^. The upper chamber of the insert was seeded with 6 × 10^4^ cells in their conventional growing medium (containing 5% FCS). The lower compartment was filled with DMEM supplemented with 10% FCS, thus creating a chemoattractant gradient. Cells were let to invade for 24 h at the 37 °C and 5%-CO2 incubator. Cells that had invaded and were adherent to the lower side of the insert were stained with DAPI. Cells were manually counted on the whole area of the insert membrane. Assays were performed in triplicate in each separate experiment.

### Epifluorescence imaging

Cells were cultured for 24–48 h on glass coverslips, then were then washed twice in PBS, before being fixed with 3.7% ice-cold paraformaldehyde in PBS. Cell permeabilization was obtained using a solution containing 50 mM NH_4_Cl, 1% BSA and 0.02% saponin. Saturation of epitopes was fulfilled by incubating 2 h with a solution containing 3% BSA and 3% Normal Goat Serum (NGS). Epifluorescence microscopy was performed with a Nikon TI-S microscope. Images were analysed using both NIS-BR software (Nikon, France) and ImageJ^©^ software 1.38I (http://rsbweb.nih.gov/ij). Fluorescent probes and conjugated antibodies were purchased from Fisher Scientific (France).

### Scanning electron microscopy

Cells were fixed by incubation for 24 h in 4% paraformaldehyde, 1% glutaraldehyde in 0.1 M phosphate buffer (pH 7.2). Cells were then washed twice in phosphate-buffered saline (PBS), and post-fixed by incubation with 2% osmium tetroxide for 1 h. Samples were dehydrated by the incubation in a series of ethanol solutions, then dried in hexamethyldisilazane (HMDS, Sigma, USA). Cells were coated with 40 Å platinum, using a GATAN PECS 682 apparatus (Pleasanton, USA), and were analysed by scanning electron microscopy (Zeiss Ultra plus FEG-SEM, Germany). A circularity index was calculated from pictures as being 4π·Area/Perimeter^2^. An index value of “1.0” indicates a perfect circle, while a value approaching “0” corresponds to an increasingly elongated shape.

### Statistical analyses

Data were displayed as box plots indicating the first quartile, the median, and the fourth quartile, whiskers indicating the minimal and maximal values, and square dots indicating the means. Non-parametric statistical tests were performed (Mann-Whitney rank sum test), using SigmaStat 3.0 software (Systat software Inc.) and P values are indicated on figures. NS stands for “not statistically different”.

## Supplementary information

Dataset1
