# Supplementary material for: Sodium Channel Nav1.5 Controls Epithelial-to-Mesenchymal Transition and Invasiveness in Breast Cancer Cells Through its Regulation by the Salt-Inducible Kinase-1
Source: Sci Rep. 2019 Dec 9;9:18652. doi: 10.1038/s41598-019-55197-5 (PMC6901527; doi:10.1038/s41598-019-55197-5)
Supplement: Supplementary file 1 — Dataset1 [file 41598_2019_55197_MOESM1_ESM.pdf]

Supplementary Figure 1

SODIUM CHANNEL *Na$_V$1.5* CONTROLS EPITHELIAL-TO-MESENCHYMAL TRANSITION AND INVASIVENESS IN BREAST CANCER CELLS THROUGH ITS REGULATION BY THE SALT-INDUCIBLE KINASE-1

Frédéric GRADEK[1*], Osbaldo LOPEZ-CHARCAS[1*], Stéphanie CHADET[1], Lucile POISSON[1,2], Lobna OULDAMER[2,3], Caroline GOUPILLE[2,3], Marie-Lise JOURDAN[2,3], Stéphan CHEVALIER[2], Driffa MOUSSATA[1,3], Pierre BESSON[2§] & Sébastien ROGER[1,4§]

[1] EA4245 Transplantation, Immunologie, Inflammation ; Université de Tours, France
[2] Inserm UMR1069, Nutrition, Croissance et Cancer ; Université de Tours, France
[3] CHRU de Tours, France
[4] Institut Universitaire de France, Paris, France

*, these authors contributed equally as co-first authors
§, these authors contributed equally as last authors

Correspondence should be addressed to:
Dr. Sébastien Roger, EA4245 Transplantation, Immunologie, Inflammation,
10 Boulevard Tonnellé, 37032 Tours, France,
Tel : (+33) 2 47 36 61 30,
Email: sebastien.roger@univ-tours.fr

Running head: *Na$_V$1.5 promotes EMT in breast cancer cells*

Supplementary Figure 1:

Representative currents recorded in three different MCF7 cells each treated for 72h with TGF-β1 (5 ng/mL) using the whole-cell configuration of the patch clamp technique. Depolarizing 16-ms pulses from -95 to +60 mV in 5-mV steps were applied every 2s from a holding potential of —100 mV. Transient inward currents were recorded in the two first examples, but not in the third one.

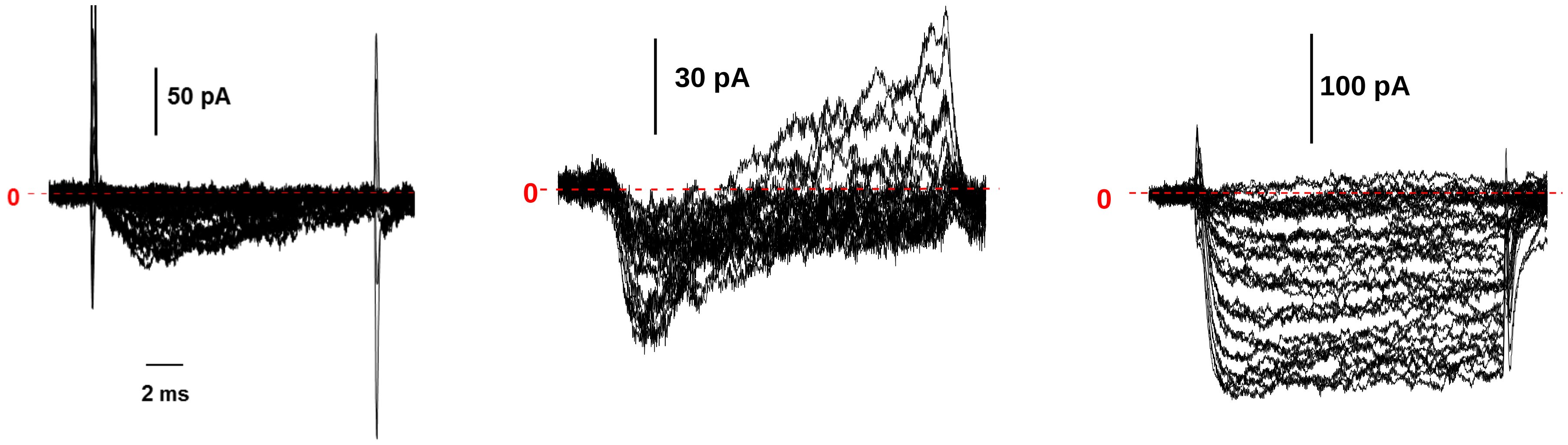

50 pA
0
2 ms
30 pA
0
100 pA
0
